# Host microbiome in tuberculosis: disease, treatment, and immunity perspectives

**DOI:** 10.3389/fmicb.2023.1236348

**Published:** 2023-09-22

**Authors:** Archana Pant, Bhabatosh Das, Gopalakrishnan Aneeshkumar Arimbasseri

**Affiliations:** ^1^Molecular Genetics Lab, National Institute of Immunology, New Delhi, India; ^2^Functional Genomics Laboratory, Infection and Immunology Division, Translational Health Science and Technology Institute, Faridabad, India

**Keywords:** tuberculosis, gut microbiota, micronutrients, anti-tuberculosis drugs, immune dynamics, drug resistance

## Abstract

Tuberculosis (TB), an airborne pulmonary disease caused by *Mycobacterium tuberculosis* (*M. tb*), poses an unprecedented health and economic burden to most of the developing countries. Treatment of TB requires prolonged use of a cocktail of antibiotics, which often manifest several side effects, including stomach upset, nausea, and loss of appetite spurring on treatment non-compliance and the emergence of antibiotic resistant *M. tb*. The anti-TB treatment regimen causes imbalances in the composition of autochthonous microbiota associated with the human body, which also contributes to major side effects. The microbiota residing in the gastrointestinal tract play an important role in various physiological processes, including resistance against colonization by pathogens, boosting host immunity, and providing key metabolic functions. In TB patients, due to prolonged exposure to anti-tuberculosis drugs, the gut microbiota significantly loses its diversity and several keystone bacterial taxa. This loss may result in a significant reduction in the functional potency of the microbiota, which is a probable reason for poor treatment outcomes. In this review, we discuss the structural and functional changes of the gut microbiota during TB and its treatment. A major focus of the review is oriented to the gut microbial association with micronutrient profiles and immune cell dynamics during TB infection. Furthermore, we summarize the acquisition of anti-microbial resistance in *M. tb* along with the microbiome-based therapeutics to cure the infections. Understanding the relationship between these components and host susceptibility to TB disease is important to finding potential targets that may be used in TB prevention, progression, and cure.

## 1. Introduction

Tuberculosis (TB) is a deadly, airborne infectious disease caused by the acid-fast bacterium *Mycobacterium tuberculosis (M. tb*), and even after years of research, it still remains a major challenge to health globally. Nearly one-third of the world's population is infected with *M. tb*. However, most individuals do not develop active tuberculosis as some clear the infection and in others the bacteria can remain dormant in the host for a very long period of time; ~5–10% of the infected individuals develop active diseases (Emery et al., 2021; Global Tuberculosis Report, 2022). Immune deficiency and malnutrition are potential risk factors of host that enhance the rate of active disease development (Global Tuberculosis Report, 2022).

Individual's microbiota plays an important role in maintaining health and modulating treatment outcomes of various diseases. It is now well-established that gut microbiota (GM) has a complex and bidirectional relationship with its host. The GM regulates host's health by playing a significant role in various biological functions, such as immunity and inflammation, digestion and absorption of nutrients, bioavailability of vitamins and other micronutrients, hunger regulation, and metabolic homeostasis (Macpherson and Harris, 2004; Larsen et al., 2010). The intimate, symbiotic, and homeostatic coexistence of gut commensals with the humans entails an effective host immune system to uphold tolerance toward innocuous stimulants and restricting overuse of host resources (Macpherson et al., 2005; Dethlefsen et al., 2007; Chu and Mazmanian, 2013). Colonization happening during early stages of life is an important event of educating and maturing the host immune system (Gensollen et al., 2016). Up to ~2.5 years of age, the intra- and inter-individual microbiota variability is highest; then, it acquires a more stable adult-like composition (Russell et al., 2012; Yatsunenko et al., 2012; Backhed et al., 2015; Caballero-Flores et al., 2019; Zheng W. et al., 2020). Approximately 40% of the entire lymphocytes of the human body are situated in the gut, and changes in GM modulate gut inflammation, highlighting the importance of microbiota-immune system interactions (Grice and Segre, 2012). In addition, the altered composition and functions of microbiota are a key feature among individuals with different health conditions, such as obesity, type 2 diabetes, blood pressure, irritable bowel syndrome (IBS), and inflammatory bowel disease (IBD) (Hou et al., 2022; Vijay and Valdes, 2022). Studies support that *M. tb* infection causes the shift in gut microbiota (Hu et al., 2019a,b; Eribo et al., 2020; Wang et al., 2021). Moreover, the antibiotics used to treat infections and illnesses also affect the gut microbial composition and functions (Table 1) (Maji et al., 2017; Wang et al., 2020; Pant et al., 2022; Patangia et al., 2022; Dahiya and Nigam, 2023).

**Table 1:** Recent studies showing association of tuberculosis infection and its treatment with gut microbiota.

**Study location**	**Subject type**	**Study details**	**Results**	**References**
India	Mice	Mice treated with broad spectrum antibiotics, followed by *M. tb* infection and then FMT.	Mice treated with antibiotics showed higher *M. tb* burden in lungs and dissemination in spleen and liver. Depletion of *Lactobacillus, Bifidobacterium, Bacteroides, Campylobacter* and increased *Enterococcus* in treated mice.	Khan et al., 2016
India	Human	6 patients and 6 HCs (close contacts). Stool collected from patients before treatment initiation, 1 week and 1 month during treatment.	SCFA producing bacteria, like *Faecalibacterium prausnitzii, Eubacterium rectale*, and *Roseburia inulinivorans* increased in patients. *Prevotella* and *Bifidobacterium* were enriched in HCs. Amino acids biosynthesis and vitamin metabolism reduced in TB subjects.	Maji et al., 2017
USA	Mice	Mice infected with *M. tb* followed by treatment with ATT drugs for up to 4 months.	ATT treatment depleted *Butyricicoccus, Ruminococcus, Acetivibrio, Peptococcus* and *Alkaliphilus* while *Erysipelatoclostridium* was increased.	Namasivayam et al., 2017
Haiti	Human	19 TB patients on treatment, 19 TB cured and 75 controls. 3 TB patients were on treatment for more than 6 months. Controls were divided into 50 IGRA negative and 25 IGRA positive (LTBI).	*Fusobacterium, Clostridium*, and *Prevotella* enriched whereas *Coprococcus, Puminococcus, Lactobacillus* and *Bifidobacterium* depleted in treatment cases. *Bacteroides* reduced, while *Ruminococcus, Faecalibacterium* and *Eubacterium* were enhanced in TB cured individuals. Therapy associated dysbiosis persisted for at least 1.2 years.	Wipperman et al., 2017.
China	Human	37 TB patients and 20 HCs. TB patients were divided into NTB (new TB cases and less than 1 week ATT) and RTB (previously treated and cured prior to becoming culture positive).	Bacteroidota reduced while Actinomycetota and Pseudomonadota enhanced in RTB Reduced *Lachnospira* and *Prevotella* genus in NTB and RTB.	Luo et al., 2017
France	Mice	Mice treated with broad spectrum antibiotics, infected with *M. tb* followed by FMT.	Bacteroidota and Bacillota reduced, Pseudomonadota enhanced in antibiotics treated mice. Microbiota dysbiosis resulted in an enhanced early colonization of *M. tb* in the lungs during first week of infection.	Dumas et al., 2018
China	Human	46 PTB subjects and 31 HCs. PTB patients before anti-TB treatment initiation.	*Clostridium bolteae* and an unclassified *Coprobacillus* were enriched in patients. *Hemophilus parainfluenzae, Roseburia hominis*, and *R. inulinivorans* discriminated patients from controls SCFA producers enhanced in HCs compared to PTB subjects.	Hu et al., 2019a
China	Human	18 pediatric TB patients and18 healthy children.	Reduced microbiota diversity, enhanced abundance of *Enterococcus, Prevotella*, and decreased abundance of *Bifidobacteriaceae, Ruminococcaceae*, and *F. prausnitzii*.	Li et al., 2019
Canada	Mice	Mice pretreated with INH/PYZ or RIF followed by H37Rv infection. Control mice were infected with H37Rv without antibiotic treatment.	RIF reduced Bacillota and enhanced Verrucomicrobia and Bacteroidetes abundance. Significant difference in Clostridia IV and XIV were observed following INH/PYZ treatment.	Khan et al., 2019
China	Human	61 TB patients, 10 LTBI and 13 HCs. Among TB cases 28 ATB, 13 and 10 TB patients on 1 and 2 weeks ATT, respectively and 10 TB cured individuals.	Minor changes during *M. tb* infection mainly the genus Bacteroides. Reduced *Ruminococcus* and *Fecaelibacterium* belonging to Clostridiales and enhanced *Bacteroides* during ATT.	Hu et al., 2019b
Tiawan	Human	25 ATB, 32 LTBI and 23 HCs.	Differences in Bacillota: Bacteroidota ratio between groups.	Huang et al., 2019
China	Human	6 patients on MDR-TB treatment, 18 patients recovered from MDR-TB and 52 TB untreated controls.	Microbe richness decreased in treated and recovered group vs untreated controls. Treated group had more Bacteroidota reduced Bacillota and Actinomycetota. 17 bacterial biomarkers identified in response to MDR-TB treatment, and 58 biomarkers identified between the MDR-TB recovered group and untreated group. Recovered group showed higher LDLC and TC compared to untreated group.	Wang et al., 2020
India	Mice	CT: control mice; Abx: broad spectrum antibiotics treated mice; Mtb: mice infected with *M. tb*; Abx-Mtb: Abx treated mice before infection, mice infected by *M. tb*; Mtb-INH: mice infected by *M. tb* and treated with INH; Abx-Mtb-INH: Abx treated mice infected with *M. tb* prior to INH therapy.	Abx treatment reduced the abundance of commensals *Bifidobacterium, Campylobacter*, and *Lactobacillus*, and enhanced the abundance of *Bacteroides and Enterococcus*.	Negi et al., 2019
China	Human	94 treatment-naive TB patients and 62 HCs.	37 ASVs more abundant in antibiotic-free TB patients with *Enterococcus, Clostridioides*, and *Rothia* presenting the highest fold changes. Increased abundances of the nucleotide metabolism pathway in the antibiotic-free TB group compared to HCs. Compared to the antibiotic-free TB group, antibiotic-exposure TB group, had a reduced abundance of *Kluyvera, Roseburia*, and *Citrobacter* and an increased abundance of *Corynebacterium, Epulopiscium*, and *Atopobium*.	Shi et al., 2021
China	Human	56 PTB cases, 36 LTBI cases and 50 HC, with no prior drug exposure for at least 1 month.	Relative abundance of *Bifidobacterium* and *Bacteroides* enhanced, while *Roseburia* and *Faecalibacterium* reduced in PTB compared to LTBI and HC. *Turicibacter, Weissella, Butyricicoccus, SMB53, Lachnospira, and Veillonella* distinguished PTB from HC (AUC: 0.82). *Lachnospira, Turicibacter, Lactobacillus*, and *Actinomyces* distinguished PTB from LTBI (AUC: 0.87). *Lachnobacterium, Akkermansia, Lactobacillus, and Bulleidia* discriminated LTBI from HC (AUC: 0.87).	Wang et al., 2022
China	Human	69 PTB untreated subjects and 63 HCs.	Relative abundance of *Bacteroides, Parabacteroides* and *Veillonella* enhanced, while *Bifidobacterium, Faecalibacterium, Agathobacter* and *CAG-352* reduced significantly in the PTB subjects. The six combined genera, including *Lactobacillus, Faecalibacterium, Roseburia, Dorea, Monnoglobus* and *[Eubacterium]_ventriosum_group* might be a set of diagnostic biomarkers for PTB (AUC = 0.90). 7 biosynthesis pathways (polyketide, ubiquinone, siderophore, lipopolysaccharide, folate, N-glycan and steroid hormone), 5 metabolism pathways (lipoic acid, taurine, ascorbate, fructose and biotin) and the degradation of different substrates, such as amino acid and glycan were increased in patients.	Ye et al., 2022

FMT, Fecal Microbiota Transplant; HC, Healthy Control; SCFA, Short chain fatty acid; ATT, Anti-TB treatment; PTB, Pulmonary TB; INH, Isoniazid; PYZ, Pyrazinamide; RIF, Rifampicin; ATB, Active TB; LDLC, Low-density lipoprotein cholesterol; TC, Total cholesterol; MDR-TB, Multi-drug resistant TB.

This review elucidates the structural and functional shift of GM upon *M. tb* infection and during the course of anti-TB treatment (ATT). This shift may result in differential metabolites and immune cells and the crucial mediators of gut–lung cross-talk via circulation. The functional capacity of GM also includes the genes required for synthesis or utilization of various macro/micronutrients which are further made available for host's utilization. Hence, dysbiosis of GM can have a major impact on host health by influencing the individual's response to ATT and treatment outcomes. Patient's compliance to treatment and complete cure from the disease are essential to curb *M. tb* dissemination and burden of antibiotic resistance. Therefore, gaining an understanding of the interplay between TB, ATT, and GM can help us discover the key players important for better treatment outcomes, new therapeutic strategies to treat TB, and reduce the treatment duration. Here, we have updated the current understanding of structural and functional composition of GM during TB infection and treatment and changes in nutrient profile and immune cell dynamics that are simultaneously happening in the patient's body. We also discuss the probable microbiome-based therapeutics to cure the TB disease along with drug resistance acquisition in *M. tb*.

## 2. Composition and diversity of gut microbiota during TB and TB treatment

### 2.1. Composition and diversity of gut microbiota during TB

**Figure 1:**
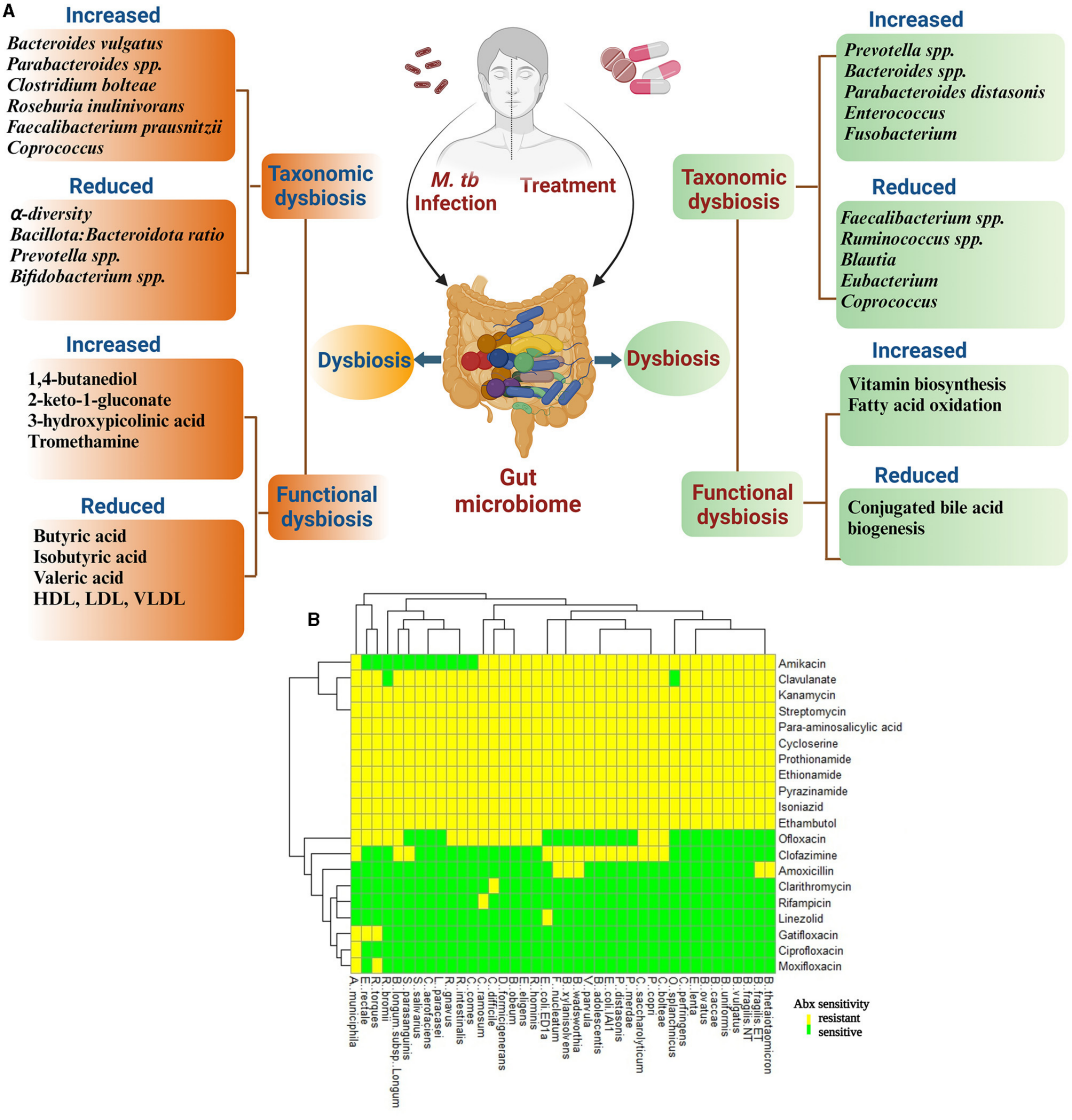
Diagram representing an overview of the effect of *M. tb* infection and anti-tuberculosis treatment (ATT) on structural and functional components of gut microbiota in TB patients. **(A)** During TB infection, there is an alteration in gut microbiota leading to reduced alpha diversity and Bacillota: Bacteroidota ratio. In this figure, the altered microbial structure resulted in differential functional features compared with healthy controls (HCs) during TB infection and untreated TB patients/LTBI as controls during treatment. Several studies have reported change in microbial composition and differential functional features upon *M. tb* infection and treatment initiation for TB; however, the results differ between studies. The reason for this may be differences in geographical locations to conduct the study, food habits, and choices of controls recruited (Ref: Maji et al., 2017; Wipperman et al., 2017; Hu et al., 2019a,b; Wang et al., 2020, 2021). **(B)** *In vitro* analysis of effect of various ATT drugs on 40 gut commensal isolates. Heatmap according to sensitivity or resistance of each strain to the respective antibiotic. Heatmap is replotted from the study by Maier et al. (2021), specifically for ATT drugs. Clavulanate is included as it is given along with Amoxicillin.

Multiple studies have analyzed the changes in gut microbiota of TB patients before the initiation of antibiotic treatment to understand the dysbiosis caused by *M. tb* infection (Figure 1A). A recent study by Wang et al. involving 83 newly diagnosed pulmonary TB (PTB) patients and 52 healthy controls (HC) showed that the PTB patients had reduced alpha diversity of the GM than the HCs. The five predominant phyla, namely Bacillota, Bacteroidota, Actinomycetota, Fusobacteria, and Verrucomicrobia, were significantly different between the two groups. The PTB group had lower Bacillota to Bacteroidota ratio with a significantly higher abundance of Bacteroidaceae, Fusobacteriaceae, Erysipelotrichaceae, and Tannerellaceae families and a significantly reduced Bifidobacteriaceae, Ruminococcaceae, Lachnospiraceae, Marinifilaceae, Barnesiellaceae, and Eggerthellaceae families compared with the HCs. In addition, the PTB group had differential abundances of 102 [with 10 dominant (≥mean 1% abundance in either group) and 92 less dominant] genera. The genera *Parabacteroides, Bacteroides, Lachnoclostridium*, and *Fusobacterium* were markedly enhanced in the PTB group, while *Roseburia, Blautia*, unidentified *Ruminococcaceae, Bifidobacterium, Romboutsia*, and *Fusicatenibacter* were enhanced in the HC group. With the linear discriminant analysis (LDA) score, the enriched taxa of the PTB group were Prevotellaceae, Bacteroidales, and *Bacteroides vulgatus* and that of the HC group were Bacillota, Lachnospiraceae, Clostridiales, Ruminococcaceae, Bifidobacteriales, Actinobacteria, and Blautia (Wang et al., 2021).

Another study with 77 samples (C = 31 and *P* = 46) also suggested significant decrease in species along with markedly low alpha diversity in TB patients than in the controls. In this study, the two groups had 25 differentially abundant species out of which only two, *Clostridium bolteae* and an unclassified *Coprobacillus* bacterium, were found enriched within the patients. However, when they analyzed the distribution of single nucleotide polymorphisms (SNPs) within the protein-coding regions of *B. vulgatus*, which was more prevalent in both the groups, 46 SNPs were differentially abundant between the groups. Interestingly, eight of these SNPs were only carried in seven carbohydrate metabolism genes including those encoding α-N-arabinofuranosidase, β-galactosidase, α-1, 2-mannosidase, rhamnogalacturonide degradation protein, rhamnulokinase, D-lactate dehydrogenase, and β-galactosidase, suggesting an altered carbohydrate preference in the gut bacteria of TB patients (Hu et al., 2019a). A study by Yongfeng et al. also found a reduction in alpha diversity in individuals with active TB infection and latent TB infection (LTBI) compared with HCs. However, the magnitude of the changes was minimal (Hu et al., 2019b).

In contrast to the above studies which were performed in China, a longitudinal study by Maji et al. on 6 TB subjects from India with three sample collection time points—before treatment (TBZ), 1 week after treatment (TBW), and 1 month after treatment (TBM)—along with 6 HCs (healthy blood relatives, with one sample collection time point) showed a higher species richness and diversity among the TB-associated microbiota. The three major phyla included Bacteroidota, Bacillota, and Pseudomonadota, which contributed >97% of total population to the Indian cohort. These patients were highly enriched in genera such as *Roseburia inulinivorans, Coprococcus, Faecalibacterium prausnitzii, Pseudobutyrivibrio, Phascolarctobacterium succinatutens*, and *Bacteroides* along with an increase in pathobionts such as *Shigella sonnei, Escherichia coli, Streptococcus vestibularis*, and *S. pneumoniae*. However, *Prevotella* and *Bifidobacterium* were markedly reduced in TB patients. Interestingly, this study also reported a significant abundance of *M. tb* in the fecal samples of TB patients along with the presence of *Mycobacterium* spp. in HC samples (Maji et al., 2017). The low sample size was a drawback of this study.

In general, these studies clearly indicate that *M. tb* infection alters the alpha diversity of GM, with the nature of changes probably dictated by the geographical location and eating habits of the population examined. To better understand the effect of microbiota on TB disease progression, it is also important to consider the genetic variation within species between different study groups.

### 2.2. Composition and diversity of gut microbiota during and after TB treatment

Antibiotic treatments are well-known to affect gut bacteria and induce dysbiosis (Figure 1B). Several recent studies suggest that TB treatment leads to prolonged taxonomic, metagenomic, and biochemical consequences via perturbation of the GM (Figure 1). A study was conducted to understand the structural changes in the GM of MDR-TB treatment and recovered groups along with two MDR-TB untreated control groups. The study suggested that the microbiota richness, measured by abundance-based coverage estimator (ACE) index, was lower in the treated and recovered groups compared with the corresponding untreated TB control groups. However, the number and evenness of observed operational taxonomic units (OTUs) were significantly higher in the recovered group, with no significant change in the treatment group compared with their respective untreated patient groups. Six phyla were identified as biomarkers to differentiate treated (Bacillota, Bacteroidota, and Actinomycetota) and recovered (Cyanobacteria, Bacteroidota, and Patescibacteria) groups from the untreated patients. During treatment, Bacillota and Actinomycetota had shown reduced relative abundance which was recovered upon discontinuing the treatment; however, Bacteroidota, which increased in response to treatment, decreased but without returning to the pre-treated level after the treatment ceased. The relative abundance of Cyanobacteria and Patescibacteria remained unchanged to the treatment group; however, they had marked reduction after recovery. Data analysis at a higher resolution identified 17 bacterial genera (16 were reduced) as biomarkers in response to the treatment, and 58 bacterial biomarkers (30 were enhanced and 28 were reduced) were identified for the recovered group (Wang et al., 2020).

Another cross-sectional study from China examined the effects of anti-TB therapy on GM in active TB patients, patients with 1 week of front-line drugs (T1), and patients with 2 weeks treatment (T2) and TB cured (Tc) groups. Treatment initiation resulted in significant microbial diversity loss compared with the active TB group. T1 group had statistically higher observed number of OTUs than the T2 and Tc groups, but no significant differences were observed in the Shannon and Pielou's evenness indices. However, the T2 and Tc groups did not show a significant difference in the above three parameters, suggesting a diversity imbalance within 2 weeks after anti-TB therapy (ATT), which persisted even in cured patients. The Non-Metric Multidimensional Scaling (NMDS) analyses have shown the formation of extreme separate cluster of active TB patient's samples compared to patients on ATT and cured TB patients demonstrating a significantly altered diversity and community structure and composition of GM upon ATT exposure. The study also determined the taxa that were markedly affected by the ATT. The taxa belonging to order Clostridiales of phylum Bacillota, namely *Faecalibacterium* (OTU 15), *Ruminococcus gnavus, and Ruminococcus* sp. *39BFAA*, were markedly affected by the therapy. However, members belonging to phylum Bacteroidota, namely *Bacteroides* (OTU230), *B. plebeius, B. fragilis, B. coprophilus, Bacteroides* (OTU 1513), *B. caccae*, and *Parabacteroides distasonis*, were markedly enhanced in the therapy group. OTU8 and OTU2972, belonging to Erysipelotrichaceae and *Enterococcus*, respectively, were markedly enriched in the T1 group compared with naive TB patients. There was a marked reduction in the genus *Bifidobacterium* in the T1 group which was significantly enhanced in the Tc group compared with the TB group (Hu et al., 2019b). Another study by Maji et al., in the PTB patients, showed that a week of ATT exposure did not reduce the abundance of *M. tb* in the gut. However, after 1 month of ATT, a significant reduction in *Mycobacterium* spp. was observed with no significant recovery in relative abundances of gut bacterial species altered due to TB disease (Maji et al., 2017).

A study by Wipperman et al. also suggested that ATT modifies the microbial taxonomic composition without affecting the total diversity. Despite being on treatment for an average of 3.4 months, the overall microbiome diversity of HRZE-treated (Isoniazid+ Rifampicin+ Pyrazinamide+ Ethambutol) TB subjects did not differ from LTBI or uninfected controls. However, a significant depletion of specific bacterial taxa during ATT exposure was observed. The HRZE-treated group resulted in an enhancement of *Fusobacterium, Erysipelatoclostridium*, and *Prevotella* with a significant reduction in *Blautia, Coprococcus, Lactobacillus, Ruminococcus, Eubacterium*, and *Bifidobacterium* compared with the LTBI group. After ~1.2 years of cessation of HRZE, the gut flora of the TB cured group was found to be depleted in *Bacteroides* with an overabundance of *Eubacterium, Faecalibacterium, and Ruminococcus*. The study suggested *Phascolarctobacterium succinatutens, Enterobacter cloacae, Bilophila, Methanobrevibacter smithii*, and *Parabacteroides* as biomarkers of TB-cured individuals (Wipperman et al., 2017).

Apart from studies with patient samples, studies on mice also show altered gut microbiome after treatment with ATT drugs. Upon comparing the effects of components of ATT, i.e., rifampicin (R) and isoniazid+pyrazinamide (H/Z), both components have markedly and distinctively altered the GM. Both alpha and beta diversities were significantly altered during R treatment, while H/Z treatment had only modest effects on alpha and beta diversities. Both treatments led to non-exclusive changes in the composition of the microbiome. The study also provided a unique perspective on the effect of these antibiotics on the host immune system. GM disruption by using R treatments in the drinking water prior to *M. tb* aerosol challenge showed no changes in *M. tb* burden in the spleen and lungs, while significantly higher burden was found in both the organs in H/Z-treated mice compared with the controls. The *M. tb* burden in mice after combined treatment (R+H/Z) was similar to H/Z treatment alone suggesting that pre-treatment with H/Z but not R compromises host defense against *M. tb* infection. The effect of H/Z on the *M. tb* burden was induced by changes in the gut microbiome as fecal microbiota transplant (FMT) markedly reduced the *M. tb* burden on the level of untreated infected animals from both the spleen and lung of H/Z pre-treated mice. However, no effect of FMT was observed in the *M. tb* burden in either organ with R pre-treatment. This may suggest that the gut dysbiosis induced by H/Z treatment leads to enhanced dissemination of *M. tb* (Khan et al., 2019).

A longitudinal study by Namasivayam et al. conducted on mice had observed a slight but significant reduction in diversity at 12th week post-*M. tb* infection or post 8 weeks HRZ treatment. Members of the order Clostridiales and phyla Bacteroidota and Mycoplasmatota were differentially abundant during longitudinal analysis of gut of TB-infected mice. Except from *Alkaliphilus* increased abundance longitudinally, no other differences remained significant. Interestingly, similar to the observation by Hu et al. (2019b), they observed marked reduction in bacterial diversity in humans, which was temporary and significant only during the first 2 weeks of ATT after which alteration in bacterial composition stabilized with minimum fluctuations. Hence, treatment with first line drugs caused a transient reduction in gut microbial diversity along with persistent, fluctuating structural changes. This dysbiosis was not observed to be affected by the presence or absence of *M. tb* infection. In addition, there was no marked enhancement in the GM diversity detected 3 months post-therapy cessation, suggesting that gut dysbiosis induced by these drugs remained for long time. However, a more detailed compositional analysis suggested few notable changes with the relative levels of different genera (Namasivayam et al., 2017).

These studies suggest that the prolonged TB treatment leads to significant alteration in structural composition of GM. While the exact nature of the changes varies between studies, probably due to diversity in the cohorts studied, there are some changes that were found recurring. Almost all studies suggest a reduction in members of the phylum Bacillota, especially class Clostridia and enrichment in members of the phylum Bacteroidota, leading to lowering of the Bacillota: Bacteroidota ratio. The loss of immunologically significant bacteria may provide an opportunity of TB re-infection or infection by pathobionts present within the individual as evidenced by mouse studies where pre-treatment with H/Z led to increased *M. tb* load. Therefore, more studies are required to understand the impact of ATT on members of the phylum Pseudomonadota carrying pathobionts.

## 3. Functional potency of gut microbiota during TB and TB treatment

In recent years, the NGS technology has made our understanding better in terms of gut microbial contribution in individual's nutrition, metabolism, and immune development. Dysbiosis leads to changes in the functional domain of GM and may contribute to the outcome of various diseases including TB (Qin et al., 2010, 2012; Karlsson et al., 2013). Recent studies suggest that various factors cause altered coding capacity of GM (Figure 1A). The HRZ-treated mice had reduced capacity for carbohydrate metabolism (Namasivayam et al., 2017). The HRZE therapy changed the microbial metabolic coding capacity which included protein secretion, sugar biosynthesis, and central metabolism. Enhanced vitamin biosynthesis and fatty acid oxidation and reduced conjugated bile acid biogenesis were also observed in HRZE-treated patients (Wipperman et al., 2017). Moreover, the indole propionate produced by gut bacteria is a potent inhibitor of *M. tb* both *in vitro* and *in vivo* (Negatu et al., 2019).

**Figure 2:**
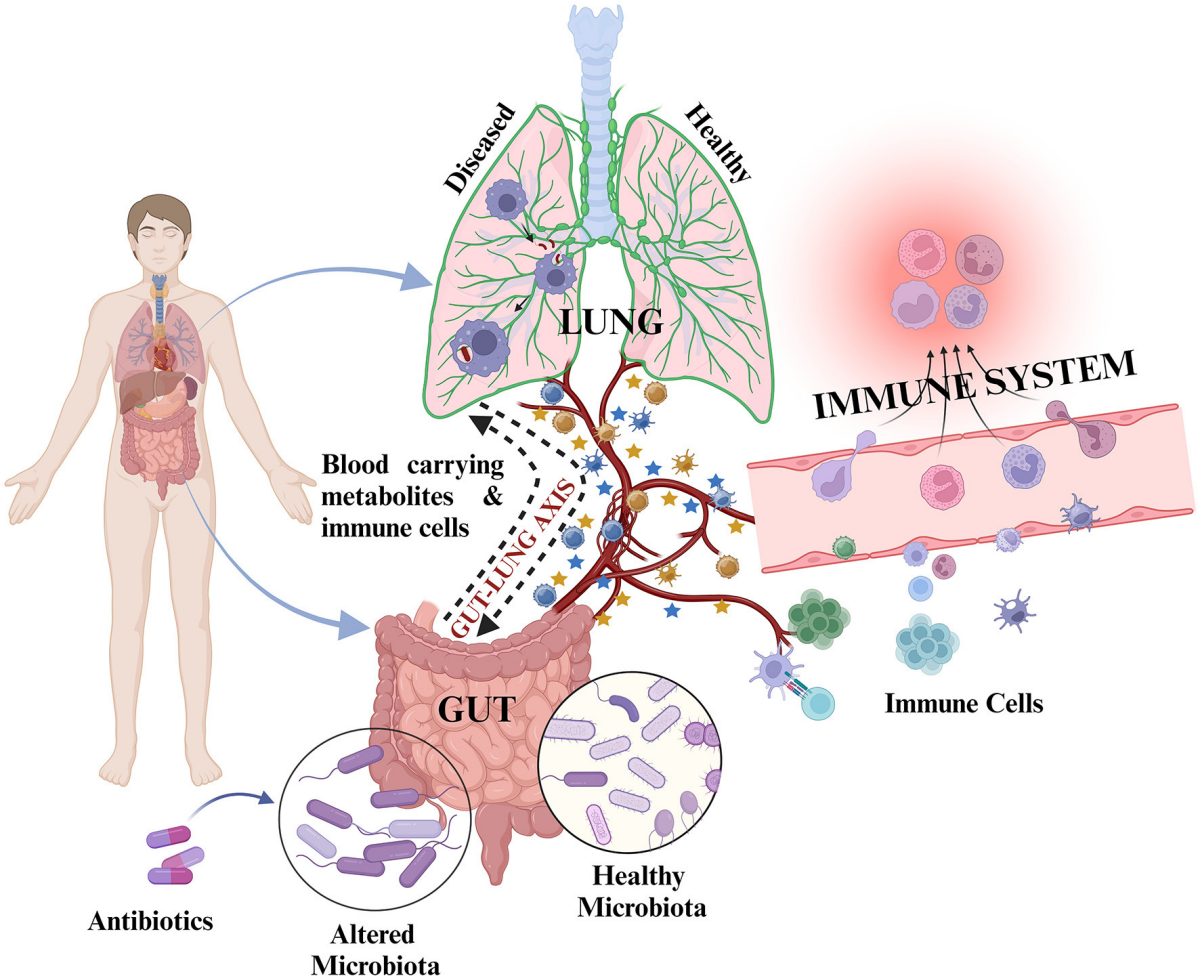
Representation of inter-organ cross-talk through the gut–lung axis: During healthy state, the cross-talk is mediated by metabolites and immune cells through circulating blood to maintain host health. Upon exposure to any infection such as *M. tb*/during diseased state/during antibiotic exposure, there is alteration in microbiota composition along with altered metabolite production and altered immune cell proliferation resulting in differential metabolites and immune cell circulation in the blood for gut–lung cross-talk. This may result in different outcomes, i.e., development or abolishment of infection and positive or poor treatment outcomes. 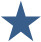 represents metabolites and 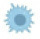 represents various immune cells. Blue colored 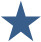 and 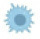 represent metabolites and immune cells during healthy state. Orange colored 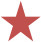 and 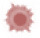 represent metabolites and immune cells during diseased state.

Structural and functional dysbiosis of GM has been linked with lung diseases through the gut–lung axis (Samuelson et al., 2015). The communication between respiratory mucosal surfaces and gut is through bidirectional movement of microbial products, cytokines, and metabolites (Figure 2). TB patient group at time-points, day 0, along with 1-week, and 1-month post-treatment initiation are significantly diverse from each other than are HCs in terms of functional repertoire of microbiota as shown by Hellinger distance calculated from KEGG Orthologs (KO) abundance. This suggested both phylogenetic and functional gut microbial dysbiosis in TB patients. In this study, many short chain fatty acid (SCFA) producers such as *Pseudobutyrivibrio, Coprococcus, Faecalibacterium*, and *Phascolarctobacterium* were significantly enhanced in the day 0 group. Interestingly, *Prevotella*, which is better equipped to consume plant-derived carbohydrates, was markedly depleted in TB patients, while the abundance of *Bacteroides* was increased (Maji et al., 2017). These two species were previously reported to show inverse correlation with the gut (Koropatkin et al., 2012; Ley, 2016; Maji et al., 2017).

In the gut of TB patients, altered relative abundance of certain genera was associated with marked changes in stool metabolites (Wang et al., 2021). The untargeted metabolic profile of TB patients identified 26 altered stool metabolites of which 17 were upregulated and 9 were downregulated. The pathways in which these metabolites were involved included ABC transporters, phosphatidylinositol signaling system, neuroactive ligand–receptor interaction, biosynthesis of primary bile acid, steroid, aminoacyl-tRNA, and amino acid, and metabolism of taurine and hypotaurine, phenylalanine, tyrosine, carbon, galactose, sulfur, inositol phosphate, ascorbate and aldarate, glycine, serine, threonine, cysteine, and methionine. *M. tb* infection reduced the SCFA production in feces which included significantly low levels of butyric acid, isobutyric acid, valeric acid, and 2-metylbutic acid. However, the levels of propionic acid and acetic acid were non-significantly reduced and elevated, respectively, in the TB group compared with HC. According to the Gini index, the models based on the combination of the top five metabolites, namely 3-hydroxypicolinic acid, 1-tetracosanol, pyrophosphate, behenic acid, and tromethamine, fairly discriminated for the TB group. These reports indicate that *M. tb* infection leads to significant changes in the metabolic profile with implications in the host metabolism.

TB patients had been found clinically to have low cholesterol level and BMI. Except for blood glucose (Glu) and albumin (Alb) levels, the serum levels of total cholesterol (Tch), high-density lipoprotein (HDL), low-density lipoprotein (LDL), and very-low-density lipoprotein (VLDL) were significantly lower in patients than in healthy controls (Deniz et al., 2007; Maji et al., 2017; Wang et al., 2021). Microbiota are well-appreciated to play an important role in the metabolic status of individuals. Four genera including *Megasphaera, Blautia, Bilophila*, and *Rhodococcus* enriched in healthy controls were positively associated with triglycerides (TG). Total cholesterol, LDL, and VLDL levels had a positive correlation with *Romboutsia, Rhodococcus, Fusicatenibacter*, and *Gordonibacter* and a negative correlation with *Weissella, Cetobacterium, Campylobacter*, and *Dorea*. HDL had a positive association with *Elusimicrobium* and *Ezakiella*, whereas *Dorea* and *Blautia* were negatively associated with glucose (Wang et al., 2021). In healthy controls, five metabolites enhanced, namely resorcinol, indoxyl sulfate, o-phosphoserine, orcinol, and inositol, had a positive association with the relative abundances of the following genera *Fusicatenibacter, Barnesiella*, unidentified *Lachnospiraceae, Dorea, Tyzzerella, Raoultibacter, Butyricicoccus, Anaerotruncus*, unidentified *Ruminococcaceae, Collinsella, Roseburia, Bilophila, and Candidatus Soleaferrea*. The TB group had 13 enhanced metabolites, namely ribonic acid, 5-aminolevulinic acid, 1,4-butanediol, taurine, 3-hydroxymethylglutaric acid, 2-keto-l-gluconate, 4-hydroxy-3-methoxyphenylglycol, 3-hydroxypicolinic acid, 4-hydroxycyclohexylcarboxylic acid, aconitic acid, hydrocinnamic acid, erythritol, and tromethamine, which were negatively associated with the following genera, *Agathobacter, Blautia, Roseburia, Anaerotruncus, Candidatus Soleaferrea, Butyricicoccus*, and *Tyzzerella* (Wang et al., 2021). Taken together, these studies indicate that *M. tb* infection and the anti-tuberculosis treatment lead to alteration of gut and systemic metabolic landscape (Figure 1A).

## 4. Lung microbiota during TB

The respiratory tract is another important organ carrying a rich microbial consortium, although smaller than gut. Compared with gut, the lung microbiota is transient and more dynamic with low bacterial burden and influenced by influx and elimination of microbiota (Beck et al., 2012; Dickson et al., 2014; Bassis et al., 2015). The upper respiratory tract (URT) consists of nasal and oral cavities which comprise diverse microbiota (Wu and Segal, 2017). Furthermore, the microbiota associated with a healthy lower respiratory tract (LRT) has the predominant phyla, i.e., Bacillota, Bacteroidota, and Pseudomonadota, similar to that detected in the healthy intestine. However, the upper respiratory tract carries 100- to 10,000-fold more bacteria than the lower respiratory tract (He et al., 2017). In healthy individuals, the lung microbiota is more similar to that of the oropharynx than of the nasopharynx (Man and De Bogaert, 2017). It is evident from these studies that the lung microbiota is crucial for immunity and provides key signals for generating and proper functioning of the immune system. Our understanding of the compositional changes of the lung microbiota in the context of TB is on an early stage. A study reported that the sputum of TB patients had significantly higher bacterial diversity than of healthy controls. In addition, the clustering pattern of PTB patients was more scattered (564/614 total genera) compared with HCs (235/614 total genera). The PTB patient's sputum is found to carry many foreign/opportunistic bacteria, such as *Mobilicoccus, Stenotrophomonas, Comamonas, Pseudomonas, Cupriavidus, Thermus, Methylobacterium, Sphingomonas, and Diaphorobacter* (Cui et al., 2012; Wu et al., 2013; Krishna et al., 2016), and is associated with disease onset, recurrence, and TB treatment failure (Wu et al., 2013). Another study reported that PTB sputum was enriched with Bacillota and Actinomycetota, whereas sputum from HCs was significantly rich in Bacteroidota and Pseudomonadota (Krishna et al., 2016). Not only the bacteria but also the microbial metabolites also influence the TB infection outcomes. Higher active TB incidence was associated with increased SCFA production by anaerobic *Prevotella* in the lower respiratory tract of HIV-infected individuals (Lawani and Morris, 2016). Butyrate prevents *M. tb* antigen-specific IFN-γ and IL-17 responses and causes increased *M. tb* antigen-specific FOXP3+ Treg cells in the lungs, hence suggesting crucial role of microbial metabolites in immune responses (Lachmandas et al., 2016; Segal et al., 2017). Moreover, there is a continuous inter-communication between the gut and lungs, the so-called gut–lung axis, which is facilitated via the blood, lymph, or directly through aspiration by the movement of bacteria or their products and inflammatory markers. It will be interesting to study whether there is gut microbiome-induced modulation of immune response within the lungs of TB patients.

## 5. Alteration of micronutrient profile during TB

**Figure 3:**
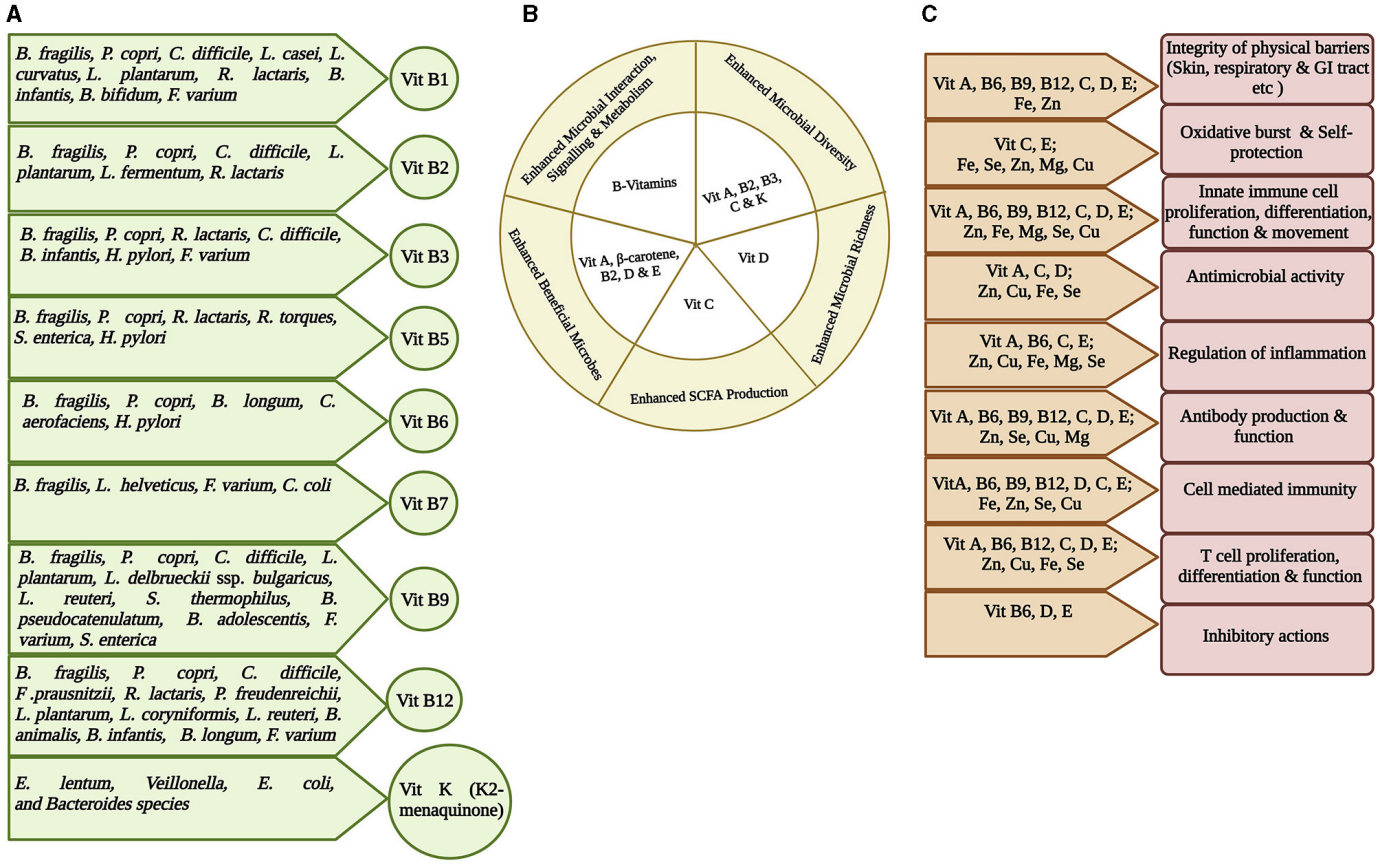
Representation of interplay among gut microbiota, micronutrients, and host immunity. Micronutrients are vital in maintaining healthy state and therefore may inhibit any infection initiation and progression. **(A)** Most vitamins, especially Vitamin B group and Vitamin K, are synthesized at some quantity within the gut by the gut bacteria. The specific gut bacteria involved in synthesizing a particular vitamin is shown (Yoshii et al., 2019; Lai et al., 2022). **(B)** The role of different vitamins in maintaining gut health by enhancing healthy microbiota richness and diversity, interaction and signaling, and SCFA production (Pham et al., 2021). **(C)** Significance of micronutrients on host immunity. Different combinations of micronutrients are required for different immune functions (Gombart et al., 2020).

Malnutrition and deficiency of micronutrients are correlated with impaired immune responses, poor prognosis, and a huge risk factor for mortality in TB patients (Figure 3) (Lubart et al., 2007; Chang et al., 2013). Nearly two-thirds of TB patients show dramatic weight reduction and malnutrition. In various studies conducted in Indonesia, India, England, and Japan, nutritional status was significantly reduced in patients with active PTB patients (Karyadi et al., 2000; Gupta et al., 2009). It is difficult to determine whether TB disease occurs due to inappropriate nutritional status leading to compromised immune status or if this condition is displayed due to emergence of disease. Nutritional deficiency in patients may also be caused by anorexia and/or hyporexia and disorders associated with restricted food intake leading to body weight reduction (Portillo and Morera, 2012). Studies have shown that deficiency in single or multiple nutrients can enhance an individual's sensitivity to infections and disease emergence (Figure 3C) (Papathakis and Piwoz, 2013). Upon comparing and analyzing the nutritional level and macronutrient and micronutrient intake of TB patients and their household contacts, both the groups presented the problem of poor nutrition. Both the groups were deficient in energy, fiber, carbohydrate, and micronutrient (vitamins and minerals) intake, with a higher lipid and protein intake. Consistent with other results, the active patients presented lower BMI than HCs (Campos-Gongora et al., 2019). The groups also showed deficiencies in minerals such as copper, iron, magnesium, calcium, and zinc (Chan and Chan, 1994; Edem et al., 2015; Campos-Gongora et al., 2019). Another study estimated that the proportion of vitamin A deficiency in TB patients and controls was 56.4% and 39.0%, respectively, and all patients and 92.5% of controls were recognized with zinc deficiency (Ghulam et al., 2009; Keflie et al., 2018). The deficiencies in vitamins such as niacin, ascorbic acid, pyridoxine, and folic acid intake were also observed (Pakasi et al., 2009). The various micronutrient levels are also inter-twined, such as zinc deficiency is associated with vitamin A deficiency by two possible mechanisms as follows: (1) the oxidative conversion of retinol to retinal: involving zinc-dependent retinol dehydrogenase enzyme, (2) the hepatic synthesis of retinol-binding protein as retinol metabolism within the liver requiring suitable zinc concentration (Christian and West, 1998; Karyadi et al., 2002). These micronutrients are well-documented to protect against TB infection (Majeed et al., 1998; Stober et al., 2001; Pakasi et al., 2009; Ali et al., 2014). Supplementation of vitamin B6 and vitamin C improved TB patients' health by preventing ATT-associated side effects (Maggini et al., 2007; Pawar et al., 2011; Miric et al., 2013). In addition, the anti-TB drugs have shown to efficiently normalize the serum levels of selenium and zinc; however, the serum vitamin D, iron, and iodine levels were not normalized by these drugs (Feleke et al., 2019). Adequate nutrition prevents the progression of active TB; hence, it is recommended to have more research studies with larger human cohorts to understand long-term consequences of malnutrition on TB. In addition, strategies aimed to promote balanced diet with adequate nutrition among the household contacts of TB patients should be promoted.

Gut microbial capability to synthesize certain vitamins, particularly vitamin K and B group, is well recognized (Figure 3A) (Rowland et al., 2018; Yoshii et al., 2019; Lai et al., 2022). *B. fragilis* and *P. copri* (phylum Bacteroidota); *C. difficile, L. plantarum, L. reuteri, L. delbrueckii ssp. bulgaricus*, and *S. thermophilus* (phylum Bacillota), some species of *Bifidobacterium spp* (phylum Actinomycetota); *F. varium* (phylum Fusobacteriota); and *S. enterica* (phylum Pseudomonadota) possess a folate biosynthesis pathway suggesting that many species in all phyla produce folate. *Bacteroides, Bifidobacterium, Streptococcus*, and *Lactococcus* spp. can synthesize folate as a common food fermentation product of carbohydrates. *Bifidobacterium* and *Lactobacillus* spp. produce 7/8 B vitamins (Kleerebezem and Vaughan, 2009). GM produces thiamin that constitutes ~2.3% of the daily human requirement (Magnúsdóttir et al., 2015). The cobalamin biosynthetic pathway is presented in 42% (110/256) of the human GM genome and can be found in all Fusobacteriota; however, it is rare in Actinomycetota and Pseudomonadota, and half of the Bacteroidota genomes are missing this biosynthetic pathway. It also acts as an immunomodulator to promote cellular immunity (Degnan et al., 2014; Magnúsdóttir et al., 2015). Another human GM study showed that 83% of bacteria (260/313 species) encode cobalamin-dependent enzymes; however, most of these 260 species lack the genes required to synthesize cobalamin (Degnan et al., 2014; Uebanso et al., 2020). The changes in gut microbiota in TB patients could significantly affect the availability and/or utilization of nutrients and may contribute to the malnutrition.

## 6. Microbiome-based therapeutics for TB

Based on the role of microbiome in modulating the host immune response and metabolism, probiotics/microbiome-based therapeutics can be a potential therapeutic component against TB. Their effectiveness against a variety of infectious diseases has been well recognized (Jia et al., 2018; Pant and Das, 2022; Tegegne and Kebede, 2022). A study has suggested *Lactobacillus crispatus* PMC201, isolated from the vaginal microbiota of healthy Korean women, as a promising probiotic against TB. The bacterium inhibited the growth of *M. tb* H37Rv strain under co-culture conditions in broth and also lowered H37Rv and XDR *M. tb* in macrophages. This bacterium did not induce dysbiosis within a human gut microbial ecosystem simulator and was non-toxic to a guinea pig model. The inhibitory effect of PMC201 on *M. tb* was due to reduced pH triggered with organic acid production by this bacteria and lower level of pro-inflammatory molecule, nitric oxide (NO), which generally increased after *M. tb* infection. The study suggests PMC201 as a potential alternate drug candidate in improvising the current TB regime (Lee et al., 2021). Another study from the same group reported the anti-TB activity of another bacterial strain, *Lacticaseibacillus rhamnosus* PMC203, derived from vaginal microbiota of healthy women. This strain also suppressed *M. tb* growth under broth co-culture condition and displayed a promising intracellular killing against both antibiotic-sensitive and resistant *M. tb*-infected murine macrophage cell lines without exhibiting cytotoxicity. It induced expression of autophagy-related gene markers in dose-dependent manner, signifying a probable intracellular killing mechanism. In this study, they used probiotic lysate instead of live bacteria, which can be used as adjuvant with TB medication without worrying about the viability in the antibiotic environment. The study suggested that beclin 1 (core protein in autophagosome nucleation) and ATG (AuTophaGy-related gene) gene complex triggers PMC203-induced autophagy to lower the TB burden on macrophage cell lines, thereby suggesting its potential to use as an anti-TB drug candidate for treating both antibiotic-sensitive and resistant TB (Rahim et al., 2022). However, both these studies have used *in vitro* co-culturing and cell line experiments that display limitation in representing PTB within patient; therefore, further studies are required to evaluate the *in vivo* efficiency and confirm the mode of action of these strains. The probiotic Nyaditum resae^®^ (Nr), having heat-killed *M. manresensis*, abolished the development of TB in murine models and patients, by enhancing the specific effector and memory Treg cell function (Cardona, 2016; Montane et al., 2017). Oral administration of *Bacteroides fragilis* promoted anti-TB immunity by increasing long non-coding RNA, lncRNA-CGB expression via the gut bacteria-lncRNA-EZH2-H3K27Me3 axis (Yang et al., 2022). *Lactobacillus plantarum* MTCC 2621 supplementation reinstated the expression of macrophage-inducible C-type lectin (mincle) on lung dendritic cells (DCs) along with enhanced anti-*M. tb* response (Negi et al., 2019). Supplementation of *L. casei* to TB patients during intensive treatment phase resulted in significant reduction in inflammatory cytokines such as TNF-alpha, IL-6, IL-10, and IL-12, along with heightened metabolites, such as maresin 1, pyridoxamine, L-saccharopine, phosphatidylserine, and phosphatidylcholine (Jiang et al., 2022). The probiotic bacteria *Lactobacillus plantarum, L. fermentum*, and *L. brevis* exhibited antimycobacterial activity in an *in vitro* study (Gavrilova et al., 2014). *Lactococcus lactis* subsp. *cremoris* MG1363 produced an anti-microbial peptide, lacticin 3147, which demonstrated its potential as a therapeutic agent by strongly inhibiting the growth of *M. tb* strain H37Ra *in vitro*, with an MIC_90_ value of 7.5 mg/L (Carroll et al., 2010). An anti-tubercular protein, NMANF2, with molecular mass of 7712.3 Da, produced by *Staphylococcus hominis* strain MANF2, has shown inhibition effect in a dose-dependent manner (Khusro et al., 2020).

Hence, to fight against the always evolving TB pathogen, identifying microbiome-based therapeutics and metabolic regulation of host could present opportunities to develop new strategies to combat drug resistance and poor treatment outcomes and reduce therapy duration.

## 7. Immune cell dynamics during *M. tb* infection

Gut microbiota is a major modulator of immune function and systemic inflammation. Th17 cells, the potent immunomodulatory effector cells, found in bulk within lamina propria of the intestine, do not appear in germ-free (GF) mice but develop upon GM establishment (Ivanov et al., 2008). Hence, host–microbiota interactions have a substantial impact on innate and adaptive immune functions. Within the gut, various components of innate immunity, such as anti-microbial peptides (AMPs) and pattern recognition receptors (PRRs), constantly interact with the microbiota, thereby shaping their composition (Ehmann et al., 2019), and play a significant role in protecting host from pathogens and maintain tissue integrity (Brown, 2006; Dethlefsen et al., 2007; Mazmanian et al., 2008; Vijay-Kumar et al., 2010; Carvalho et al., 2012; Lee et al., 2018; Erturk-Hasdemir et al., 2019; Ramakrishna et al., 2019). The microbial metabolite sensor, such as Ffar2, is known to regulate the proliferation and function of a specific group of ILCs, i.e., group 3 ILCs (Chun et al., 2019). Moreover, the microbiota-derived metabolites such as secondary bile acids modulate intestinal RORγ+ Treg cell homeostasis (Song et al., 2020) and indole, encouraging the epithelial barrier fortification by upregulating the tight junctions and associated cytoskeletal proteins (Bansal et al., 2010; Zheng D. et al., 2020). T-cell-dependent and independent IgA secretion by the B cells in response to the commensals also play key role in gut homeostasis (Peterson et al., 2007; Sutherland et al., 2016).

GM dysbiosis, leading to immune dysregulation, is directly linked with chronic respiratory disease development such as asthma, cystic fibrosis, and COPD (Fujimura and Lynch, 2015; Hauptmann and Schaible, 2016; Wang et al., 2017). However, the role of microbiota-mediated immunity during *M. tb* infection remains less explored. Studies suggest the prominent role of GM in triggering and promoting the initiation and maintenance of immune responses during *M. tb* infection. Compared with *H. pylori* seronegative individuals, the seropositive individuals with LTBI show enhanced *M. tb* antigen-specific Th1 response and IFN-γ production and were less likely to progress to active TB (Perry et al., 2013). In infants, the abundance of *Bifidobacterium* spp. in GM was found to be linked with enhanced purified protein derivative (PPD)-specific T-cell responses after BCG vaccination (Ota et al., 2002). Fascinatingly, the GM disruption altered the adaptive immune response to TB, leading to enhanced Treg cells and lowering the TNF-α and IFN-γ secreting CD4+ T cells upon *M. tb* challenge (Khan et al., 2016). A gut commensal, *B. fragilis*, directly regulates lncRNA, termed lncRNA-CGB, which is downregulated due to GM dysbiosis induced by broad-spectrum antibiotics during TB infection in mice. Interaction of lncRNA-CGB with EZH2 negatively regulated H3K27 tri-methylation (H3K27Me3) epigenetic programming, resulting in heightened IFN-γ expression and enhanced anti-TB immunity (Yang et al., 2022). In another study, the mice exposed to broad-spectrum antibiotics to induce gut dysbiosis displayed reduced expression of mincle on lung DCs. These phenotypically and functionally impaired DCs, with reduced capacity to stimulate naive CD4 T cells, were not able to curb *M. tb* survival. This immune defect was significantly improved by *in vivo* trehalose-6,6-dibehenate (TDB: mincle ligand) administration, enhancing lung DC function and T-cell response along with expansion of *Lactobacillus* (Negi et al., 2019). These studies suggest that GM is important to establish either immunity or TB infection in the lungs and strongly support the scope of microbiota-based treatments as potential future therapeutics against TB.

## 8. Development of drug resistance in *Mycobacterium tuberculosis*

Drug resistance (DR) in *M. tb* has limited the efforts to control and eradicate TB. In the next 35 years, it is predicted that ~75 million people would be killed due to DR-TB and put a cost of $16.7 trillion to the worldwide economy. Various factors for generation of DR-*M. tb* include strain's genetic background, its fitness, and capability to adapt to the niche, along with environmental and host-specific factors (Brites and Gagneux, 2015). Two factors responsible for development of DR in *M. tb* can be extrinsic and intrinsic. Extrinsic factors include the societal factors associated with TB and the quality of services provided in order to prevent and control TB. Intrinsic factors include acquiring genetic mutations (Gygli et al., 2017; Nguyen et al., 2018; Swain et al., 2020) in genes encoding for drug targets or enzymes for drug activation, which are introduced through single-nucleotide polymorphisms (SNPs) or insertion/deletions (indels). The role of horizontal gene transfer for DR acquisition is not very well informed in *M. tb* (Namouchi et al., 2012; Dookie et al., 2018). Drug concentration remains the important determining factor of mutation acquisition linked with resistance. The gut/lung microbiota carrying a huge enzymatic repertoire could play a significant role in inducing DR in *M. tb* as these antibiotics induced altered microbiota probably carrying an altered enzymatic and metabolic profile, thereby generating altered metabolites for gut–lung cross-talk. Hence, more studies should be carried out to explore and understand the microbiota-induced mechanisms in inducing drug resistance in *M. tb*, if any.

## 9. Conclusion

The understanding of the role of indigenous microbiota in infection, inflammation, and metabolic diseases and their homeostasis has substantially improved in the last two decades; however, several challenges and questions remain to be addressed (Box 1). Thorough investigations on the role of microbiota in contributing to differential inter-individual susceptibility to active TB and LTBI, reactivation from latency, and clearance with or without drugs are few, and more studies are urgently required. Another critical component is to find out whether ATT-induced GM dysbiosis could augment the susceptibility to TB reactivation after a successful cure and the emergence of drug resistance. Moreover, inter-individual variations in metabolizing dietary components are mainly due to the differential structural and functional composition of GM, which can have implications for the effects of some metabolites on health. Since, within this dynamic ecosystem, varying outcomes can result for the same substrate depending on the species existing and their proximity, therefore, apart from inspecting the microbiota in TB disease, a more in-depth understanding of GM cross-talk with airway epithelium, residing microbiota, and the innate or adaptive immune systems will be required. This understanding could transform the way TB is treated currently and may contribute to the development of novel therapeutic approaches.

Box 1 **Outstanding questions.**

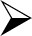
 What are the metabolic and gene expression triggers that lead to gut microbiota alteration in the TB patients even before treatment initiation?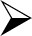 What are the changes in metabolic profile of gut microbiota in TB patients before, during, and after ATT exposure?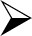 Is there any bacterium or gene that can be used as a biomarker to distinguish sensitive and resistant TB infections? Can these biomarkers be manipulated in order to re-sensitize the resistant infections?

## Author contributions

AP conceived the idea, collected the literature, and drafted the review. BD and GA edited and finalized the review. All authors contributed to the article and approved the submitted version.
